# Chronic Ultrasound (‘Emotional’) Stress in Mice Exacerbates Colitis Through GSK3β-Mediated Neuroimmune Dysregulation and Is Ameliorated by Probiotic Intervention

**DOI:** 10.3390/ijms27209092

**Published:** 2026-10-14

**Authors:** Anna Gorlova, Abrar Haque, Nathaniel Reid, Anatoly Inozemtsev, Sholpan Askarova, Aleksei Umriukhin, Anna Obleukhova, Elena Zakharova, Johannes P. M. de Munter, Alexander M. Dudchenko, Almagul Kushugulova, Alexey V. Deykin, Tatyana Cherdyntseva, Dmitrii Pavlov, Tatyana Strekalova

**Affiliations:** 1 Department of Psychiatry and Neuropsychology, Maastricht University, 6211 LK Maastricht, The Netherlandsh.demunter@neuroplast.com (J.P.M.d.M.);; 2 Research and Education Resource Center, Peoples Friendship University of Russia (RUDN University), 117198 Moscow, Russia; 3 Hotchkins Brain Institute, University of Calgary, Calgary, AB T2N 1N4, Canada; 4 Department of Anesthesiology, The Ohio State University, Columbus, OH 43210, USA; 5 Department of Biology, Lomonosov Moscow State University, 119991 Moscow, Russia; 6 Center for Life Sciences, National Laboratory Astana, Nazarbayev University, Astana 010000, Kazakhstan; 7 Department of Normal Physiology, Sechenov University, 119048 Moscow, Russiaobleukhova_a_a@student.sechenov.ru (A.O.); 8 Institute of General Pathology and Pathophysiology, 125315 Moscow, Russia; zakharova.ei.27@gmail.com (E.Z.); amdudchenko@gmail.com (A.M.D.); 9 Laboratory of Genetic Technology and Gene Editing for Biomedicine and Veterinary, National Research Belgorod State University, 308015 Belgorod, Russia; 10 Division of Molecular Psychiatry, Center of Mental Health, University of Hospital Würzburg, Magarete Höppel-Platz 1, 97080 Würzburg, Germany

**Keywords:** irritable bowel syndrome (IBS), inflammatory bowel disease (IBD), glycogen synthase kinase 3β (GSK3β), ultrasound stress, emotional stress, gut–brain axis, dextran sulfate sodium (DSS)-induced colitis, probiotics, animal model, mice

## Abstract

**Background:** Growing evidence supports a bidirectional relationship between ‘emotional’ (psychological) stress and intestinal inflammation. ‘Emotional stress’, a form of stress that is triggered by negative information and experiences of non-physical nature, is an established risk factor for the development of subclinical intestinal inflammation, called irritable bowel syndrome (IBS), to inflammatory bowel disease (IBD). However, the underlying mechanisms of this transition remain unclear, owing to the limited availability of appropriate animal models. Glycogen synthase kinase-3β (GSK3β), a molecular hub of ‘emotional stress’ response, as shown particularly in the ultrasound stress (US) model, and an important player in gut inflammation, may underlie this transition. Here, we propose a mouse paradigm for the progression from IBS to IBD-like syndrome by combining the US with dextran sulfate sodium (DSS)-induced colitis paradigm in mice and investigate the role of GSK3β signaling under these conditions. **Methods:** C57BL/6 mice were subjected to a 21-day US consisting of irradiation of alternating ‘negative’ and ‘neutral’ frequencies, followed by 5 days of 0.5% DSS exposure via drinking water. A standardized anti-inflammatory probiotic VSL#3 whose efficacy was shown in DSS-based models was administered to a subgroup of mice. Behavioral, histological, and molecular outcomes previously established in US and IBS models were evaluated. This included parameters of anxiety, anhedonia, time of intestinal transit, fecal water content, and brain and gut neutrophil infiltration, as well as gene expression of pro-inflammatory cytokines and of GSK3β, and serum levels of pro-inflammatory molecules. Given the important role of the nervus vagus in brain–gut interactions, molecular changes were studied in the nervus vagus as well. **Results:** Mice exposed to the combined US and DSS challenge had impaired intestinal transit and increased fecal water content, neutrophil infiltration, the upregulation of pro-inflammatory mediators and GSK3β in the gut that together manifested the IBD-like state. In this group, upregulated gene expression of these markers was found in the prefrontal cortex and the hippocampus, as well as in the vagus. The combined US and DSS challenge exacerbated anxiety- and anhedonia-like behaviors and elevated blood cytokine and chemokine levels. In comparison to these changes, solitary exposures to US or DSS exerted smaller effects on the majority of measured parameters. Probiotic treatment attenuated most of the physiological, inflammatory, and behavioral abnormalities induced by the combined US and DSS intervention. **Conclusions:** The mouse model employed here using both US and DSS recapitulated the progression of IBS to the IBD associated with ‘emotional stress’ and demonstrated upregulated gut and brain *Gsk3β* expression accompanying this transition. The described model provides a useful paradigm for investigating the mechanisms underlying the disease development from IBS- to IBD-like pathology.

## 1. Introduction

Among the gut disorders associated with emotional (or psychological) stress, irritable bowel syndrome (IBS) is one of the most prevalent conditions [1,2]. IBS is characterized by recurrent abdominal dysfunction and pain in the absence of structural abnormalities and overt intestinal inflammation [3,4]. Affecting approximately 10–15% of the global population, IBS significantly reduces quality of life and increases the risk of inflammatory bowel disease (IBD), which, in contrast to IBS, is manifested by tissue injury and overt inflammation, leading to even more substantial medical and societal burden [5,6]. IBD is characterized by chronic gastrointestinal inflammation, epithelial injury, immune cell infiltration, disruption of mucosal architecture, and progressive tissue damage [7,8]. Patients with IBD frequently develop severe gastrointestinal symptoms, including persistent diarrhea, abdominal pain, rectal bleeding, weight loss, and fatigue, often requiring long-term immunomodulatory therapy or surgical intervention [8,9].

Longitudinal studies report an increased incidence of IBD following an IBS diagnosis [10]. Although no linear mechanism of conversion between the two conditions has been established, emotional stress has been proposed to promote this progression [10,11,12,13]. The so-called ‘emotional’ or ‘psychological’/‘mental’ stress is defined as a condition characterized by negative emotional states, including anxiety, fear, worry, helplessness, and emotional discomfort, arising from the perception of adverse experiences and negative information in the absence of direct physical harm or organic pathology [14,15,16].

While other types of stressors in their majority are associated with negative emotions as well, such as, for example, increased signs of anxiety and depressive-like behaviors after physical stress, pain, externally induced increases in inflammatory factors or glucocorticoid levels, related states of stress are not triggered by a perception and processing of adverse information and do not involve a cognitive element as a key feature. The term ‘emotional stress’ and similar definitions are used to label a state of stress that is resulting from adversities in human life, such as death of relatives, divorce, humiliation or defeat, loss of social status, deterioration of financial status. A focus on this phenomenon as a separate form of stress, which is predominantly determined by cognitive elements, originates from the view of Epicurus, who emphasized the ‘imperturbability of mind’ (‘ataraxia’) as a key prerequisite of overcoming the external noxious influences [17]. Although other forms of stress may also contribute, psychological stress is one of the most frequent causes of this progression [2,18].

Reciprocally, patients with major depressive disorder (MDD), anxiety disorders, and other stress-related neuropsychiatric conditions frequently suffer from gastrointestinal symptoms, including abdominal pain, altered bowel habits, impaired intestinal transit, and visceral hypersensitivity, resembling IBS [2,19]. Experimental animal models of stress support compromised gut functions associated with chronic stress. For example, 10-day water avoidance stress in adult Ws/Ws rats increased intestinal permeability, bacterial adherence to the epithelium, mast cell activation, and mild gastrointestinal inflammation characterized by neutrophil and mononuclear cell infiltration [20]. Five weeks of chronic unpredictable mild stress (CUMS) in male Wistar rats increased intestinal permeability, promoted bacterial translocation into the colonic mucosa, altered gut microbiota composition, and induced colonic inflammation, including neutrophil infiltration [21].

Animal studies addressing the transition from an IBS-like to an IBD-like state and the role of emotional stress are currently scarce. Little is known about the signaling mechanisms underlying this pathological process. Social defeat and 24 h of restraint stress were shown to upregulate the intestinal expression of C-C motif chemokine ligand 2 (CCL2), which mediates the recruitment of bone marrow-derived inflammatory monocytes to the gut and is associated with barrier disruption; however, the mechanisms underlying this process remain unknown [22].

One candidate mechanistic link between emotional stress and gut inflammation is glycogen synthase kinase 3β (GSK3β), which is implicated in both stress-related neuropsychiatric disorders, including anxiety and depression [23,24,25], and intestinal inflammatory diseases [26,27]. Animal stress models have shown elevated GSK3β activity in limbic brain regions of mice and rats [25,28,29,30,31,32], and pharmacological or genetic inhibition of GSK3β exerts antidepressant- and anxiolytic-like effects in these paradigms [33,34]. The ultrasound model of ‘emotional stress’ employed here upregulates Gsk3β expression in the hippocampus, amygdala, and prefrontal cortex [23,35], and these changes are reversible with anti-inflammatory treatments [36,37]. In addition, the ultrasound mouse model of ’emotional stress’ that is based on chronic exposure of rodents to unpredictably alternating low and high frequencies at a specific range, which corresponds to vocalizations associated with ‘negative emotional state’ or ‘neutral/positive’ emotional states [38,39], exerts brain upregulation of *Gsk3β* [23,35].

GSK3β also contributes to intestinal inflammation [26,27]. In mice with DSS-induced colitis, pharmacological inhibition of GSK3β attenuates intestinal inflammation by shifting signaling from NF-κB towards CREB and reducing pro-inflammatory cytokine production [26], and comparable protection has been reported in TNBS-induced colitis in rats [27]. Conversely, DSS exposure upregulates gut *Gsk3β* in parallel with myeloid cell infiltration, cytokine induction and impaired intestinal transit [40,41]. GSK3β is thus implicated both in stress-related anxiety- and depression-like states and in the regulation of gut inflammation. However, its role in the progression from IBS to IBD has not been addressed in the available literature.

Together, a body of evidence suggests the implication of GSK3β in the neuropsychiatric mechanisms of anxiety- and MDD-like disorders related to emotional stress, as well as the important role of GSK3β-mediated regulation in gut inflammation. However, the role of GSK3β in the progression from IBS to IBD has not been addressed in the available literature. To address this gap, we sequentially applied the US paradigm of emotional stress and the IBS-like model based on the administration of low-dose DSS (0.5% in drinking water). In the previously established IBS-like model, subchronic five-day long administration of DSS 0.5% dissolved in drinking water exerts subclinical intestinal inflammation with mild epithelial and immune alterations but without overt disease, reproducing low-grade immune activation and intestinal injury [42]. In contrast, at the standard colitogenic dose of 5%, DSS increases gut GSK3β activity, which is accompanied by epithelial barrier disruption, crypt damage, goblet cell loss, mucosal ulceration, increased infiltration of CD11b^+^ myeloid cells and Ly6G^+^ neutrophils, increased myeloperoxidase (MPO) activity, and expression of inflammatory elements of NF-κB signaling [26,42].

Given the high importance of the ‘emotional’ form of stress in the etiology of human disorders, we proposed a rodent model with US of unpredictably emerging ‘emotionally negative’ sound cues may offer some analogy to major human stressors causing depression. The US paradigm of emotional stress uses prolonged exposure to unpredictable alternating ultrasonic frequencies of ‘negative’ and ‘neutral’ modalities emitted by mice in nature [15,23] in the absence of direct physical stressors. In this stress paradigm, frequencies unpredictably alternate between 20 and 25 kHz, a range associated with negative affective states in rodents, and 25–45 kHz, considered relatively neutral [38,39]. This approach is based on ethological studies showing that ultrasonic communication in rodents is frequency-dependent [39,43,44,45]: rodents emit 20–25 kHz calls under aversive conditions such as social defeat, pain, and maternal separation [46,47,48,49], whereas higher-frequency calls above 50 kHz are typically associated with positive social and reproductive inputs, including mother–pup interactions, mating, and social exploration [50,51,52,53,54] and thus were avoided. Random alternation of ultrasound frequencies associated with opposing emotional modalities is meant to reproduce a key feature of unpredictable chronic stress, an established approach for modeling MDD-like states in rodents [55,56].

The US model has been extensively validated as a model of chronic psychological stress and an MDD-like state [14,23,36,37] in which anhedonic and helpless behaviors are accompanied by increased blood glucocorticoid levels, a classic endocrine stress marker, and elevated brain and blood levels of IL-1β, IL-6, and TNF, and exacerbated oxidative stress supposedly via NF-κB and mitogen-activated protein kinase (MAPK) signaling [14,23,36,37,57,58,59]. We therefore hypothesized that cumulative changes in the regulation of GSK3β, NF-κB, and cytokines induced by combined exposure to US and administration of low-dose DSS (0.5%) can drive a progression from an IBS-like to an IBD-like state.

Therefore, C57BL/6 male mice were subjected to a 3-week US paradigm followed by 5 days of exposure to a low-dose DSS (0.5%) drinking solution. Commonly accepted manifestations of colitis in mice, including fecal water content, whole-gut transit time in the charcoal meal test [60], and colorectal transit time in the bead expulsion test [61,62], were studied. Neutrophil infiltration was studied in the gut and brain using MPO immunostaining, where the ascending, transverse, and descending colon and the rectum were investigated separately because of previous reports of regional differences in rodent models of chemically induced colitis [63,64]. In the brain, MPO staining was quantified in the key brain regions involved in stress response, such as the medial prefrontal cortex (mPFC), hippocampus, amygdala, and hypothalamus [23,57]. *Gsk3β* expression was measured by RT-PCR in the above-mentioned brain and gut regions. In this study, we also chose to investigate the gene expression of *Gsk3β* in the vagus nerve, as it provides a bidirectional neural interface between the brain and gastrointestinal tract and participates in both afferent signaling and efferent cholinergic regulation of intestinal immunity [65,66]. Finally, cytokine and chemokine levels were profiled to determine whether the combined use of US and IBS paradigms alters systemic immune activation.

Additionally, to test the sensitivity of the induced colitis state resembling an IBD-like syndrome, a subset of challenged animals received the standardized high-potency probiotic VSL#3 during DSS exposure [67,68]. This multi-strain formulation includes *Lactobacillus*, *Bifidobacterium*, and *Streptococcus* species [69] and has demonstrated efficacy in patients with colitis and IBD [70,71]. In IL-10-deficient mice with spontaneous colitis, VSL#3 restored colonic barrier integrity and reduced mucosal TNF and IFN-γ [72]. In BALB/c mice with acute colitis induced by high-dose DSS (5%) in drinking water, VSL#3 reduced histological colitis hallmarks by increasing villus height and reducing muscularis externa thickness, prevented increased epithelial permeability, preserved the tight-junction proteins occludin, ZO-1, and claudins, and reduced epithelial apoptosis [73]. In Muc2-deficient mice, which exhibit impaired colonic mucus barrier function and spontaneous colitis, VSL#3 reduced colonic proinflammatory cytokines and chemokines and improved epithelial barrier function both at baseline and after high-dose DSS (5%) challenge [74].

Mice exposed to the combined US and IBS paradigm showed impaired intestinal transit and increased fecal water content, enhanced neutrophil infiltration, elevated expression of pro-inflammatory mediators, and increased *Gsk3β* expression in both the gut and brain, accompanied by anxiety- and anhedonia-like behaviors and blood-elevated cytokine and chemokine levels. VSL#3 ameliorated most of these physiological, inflammatory, and behavioral changes. Thus, the paradigm recapitulates the stress-driven progression from a subclinical IBS-like state to an IBD-like state and identifies GSK3β as a candidate mediator of this transition.

## 2. Results

### 2.1. Gastrointestinal Dysfunction

**Figure 1:**
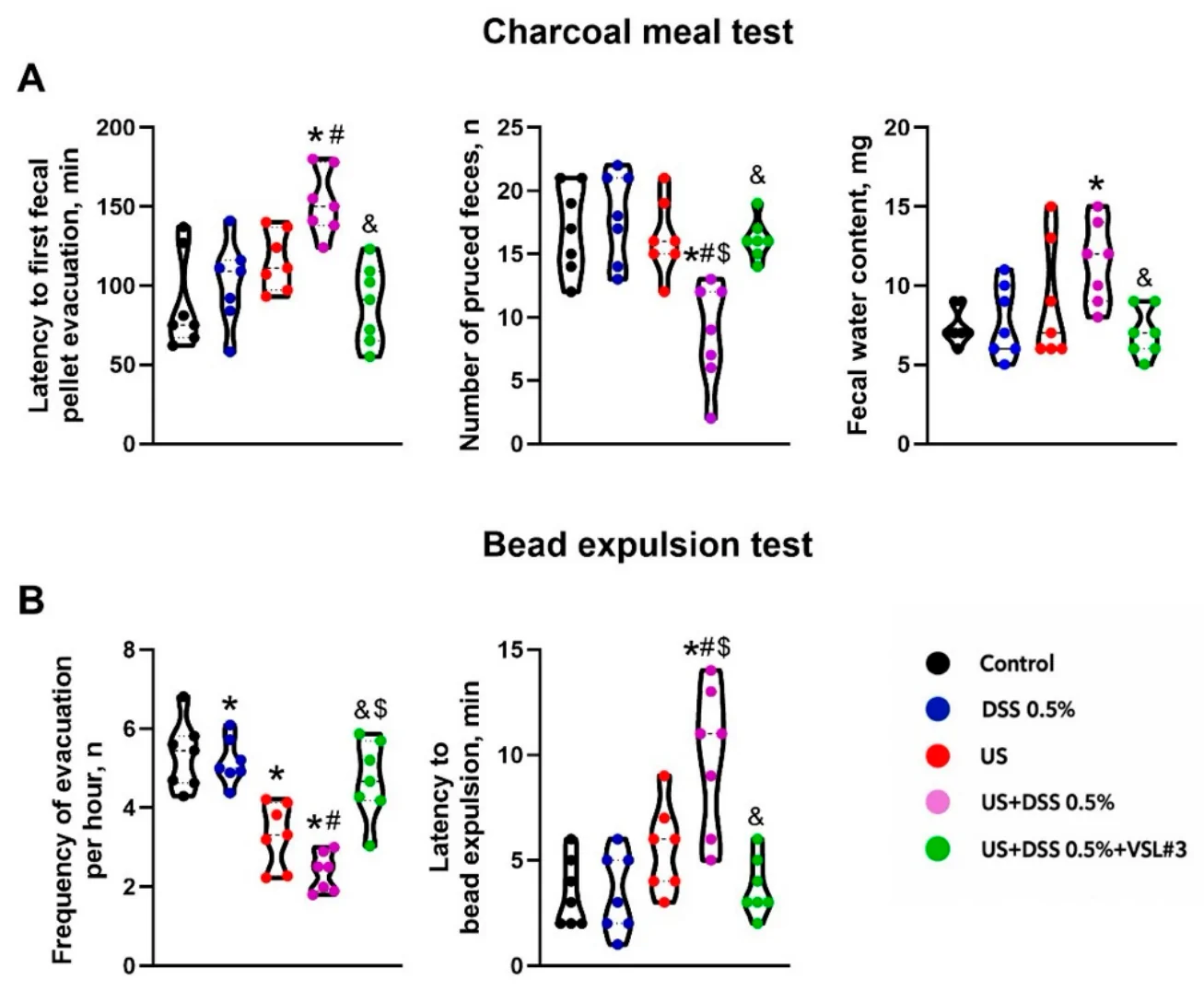
Combined challenge to US and DSS impairs gastrointestinal motility and bowel function, which are restored by probiotic treatment. (**A**) Whole-gut transit latency, fecal water content and fecal pellet output in the charcoal meal test. (**B**) Colorectal transit frequency and latency in the bead expulsion test. * *p* < 0.05 vs. control, # *p* < 0.05 vs. DSS, $ *p* < 0.05 vs. US, & *p* < 0.05 vs. US + DSS, one-way ANOVA and post hoc Tukey’s test, all n = 7. Data are shown as violin plots with individual data points. US—ultrasound, DSS—dextran sulfate sodium.

Latency to first fecal pellet evacuation in the charcoal meal test differed significantly between groups (F = 8.161, 0.0001, one-way ANOVA). This measure was significantly increased in the US + DSS group in comparison with control, DSS and US + DSS + VSL#3 groups (*p* = 0.0003, *p* = 0.0046 and *p* = 0.0003, respectively, Tukey’s test; Figure 1A). The number of produced feces differed between groups as well (F = 9.298, *p* < 0.0001, one-way ANOVA). This indicator was significantly lower in the US + DSS group compared to controls, US, DSS and US + DSS + VSL#3 groups (*p* = 0.0004, *p* = 0.0011, *p* < 0.0001 and *p* = 0.0014, respectively, Tukey’s test; Figure 1A). Significant group differences were revealed for fecal water content (F = 3.8, *p* = 0.0129, one-way ANOVA). This measure was significantly higher in the US + DSS group than in control and US + DSS + VSL#3 groups (*p* = 0.0322 and *p* = 0.0144, respectively, Tukey’s test; Figure 1A).

In the bead expulsion test, the frequency of pellet evacuation and latency to bead expulsion differed significantly between groups (F = 19.6, *p* < 0.0001 and F = 11.4, *p* < 0.0001, respectively; one-way ANOVA). Bead frequency was significantly decreased in the US + DSS group compared to control and DSS groups (*p* < 0.0001 and *p* = 0.0008, respectively, Tukey’s test); it was also significantly lower in US and DSS groups compared to control (*p* < 0.0001 and *p* = 0.0003, respectively, Figure 1B). The US + DSS + VSL#3 group had significantly elevated levels of this measure compared to the US and US + DSS groups (*p* = 0.0156 and *p* < 0.0001, respectively; Figure 1B). Latency to bead expulsion was significantly prolonged in the US + DSS group compared to the control and DSS groups (both *p* < 0.0001, Tukey’s test), as well as the US group (*p* = 0.0078). In the US + DSS + VSL#3 group, this indicator was significantly shorter than that in the US + DSS group (*p* = 0.0001; Figure 1B). A separate group of mice that received 5% DSS showed profound alterations in the charcoal meal and bead expulsion tests (see Supplementary File, Figure S2).

### 2.2. Neutrophil Infiltration into the Gut Wall Across Gastro-Intestinal Tract

**Figure 2:**
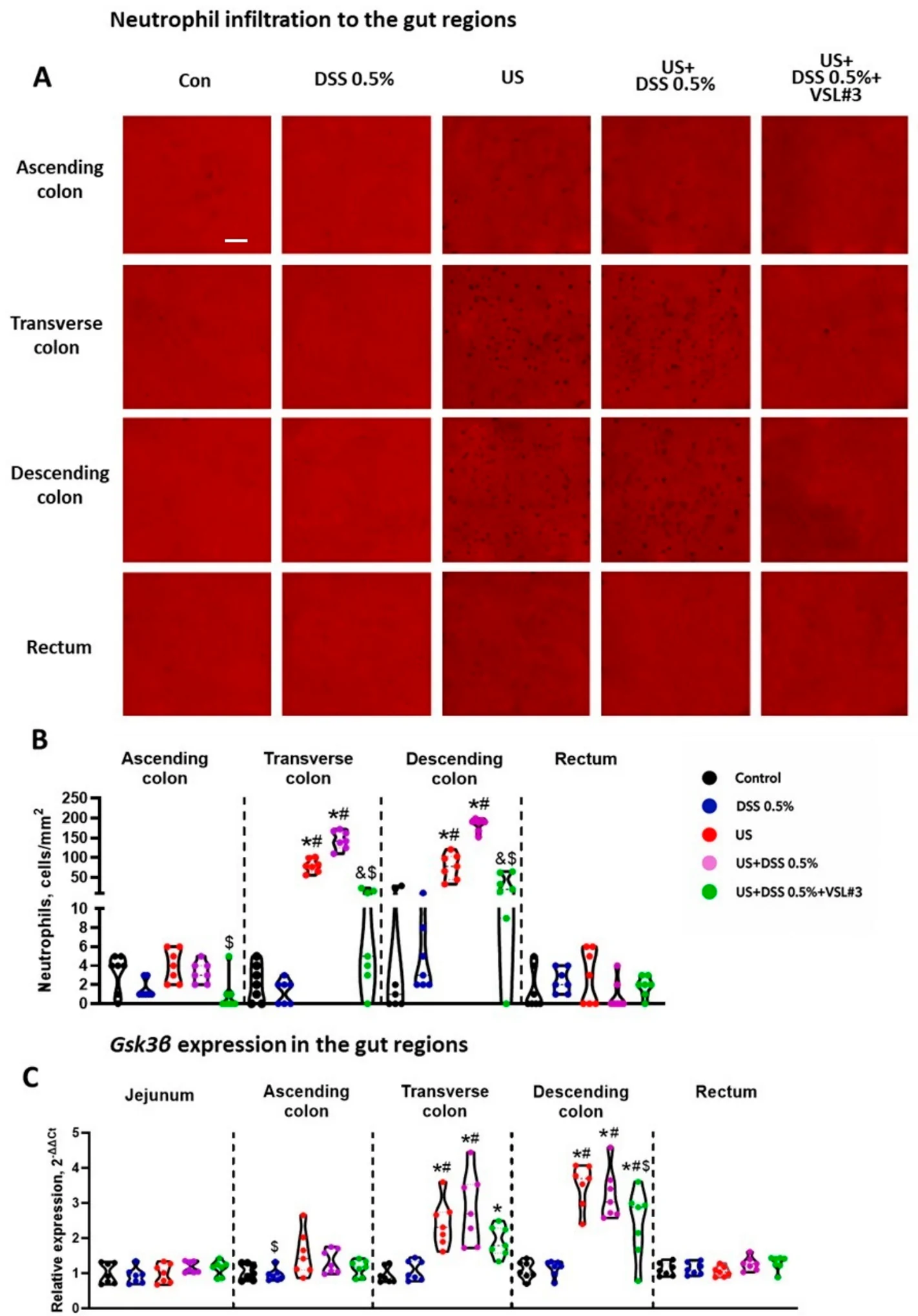
Regional neutrophil infiltration and *Gsk3β* expression are increased in the transverse and descending colon following combined exposure to US and DSS model. (**A**) Representative MPO-stained sections from the ascending colon, transverse colon, descending colon, and rectum. Dark MPO-positive cells indicate neutrophil infiltration within the gut wall. (**B**) Quantification of MPO-positive neutrophils per mm^2^ in each gut region. (**C**) Relative *Gsk3β* mRNA expression in the jejunum, ascending colon, transverse colon, descending colon, and rectum, normalized to that of the control. * *p* < 0.05 vs. control, # *p* < 0.05 vs. DSS, $ *p* < 0.05 vs. US, & *p* < 0.05 vs. US + DSS, one-way ANOVA and post hoc Tukey’s test, all n = 7. Data are shown as violin plots with individual data points. US—ultrasound, DSS—dextran sulfate sodium.

Significant group differences were found for neutrophil infiltration in the ascending colon (F = 4.586, *p* = 0.0052, one-way ANOVA). This measure was significantly lower in the US + DSS+ VSL#3 group than in the US only group (*p* = 0.0102, Tukey’s test; Figure 2A,B). Significant group differences were observed in neutrophil infiltration in the transverse colon (F = 161.7, *p* < 0.0001, one-way ANOVA). It was significantly higher in the US group than in the control, DSS, and US + DSS + VSL#3 groups (all *p* < 0.0001, Tukey’s test). It was also significantly higher in the US + DSS group than in the control, DSS, US, and US + DSS + VSL#3 groups (all *p* < 0.0001, Tukey’s test; Figure 2B). The groups differed significantly in terms of neutrophil infiltration in the descending colon (F = 91.46, *p* < 0.0001, one-way ANOVA). Similarly, this indicator was significantly higher in the US group than in the control, DSS, and US + DSS + VSL#3 groups (*p* < 0.0001, *p* < 0.0001, and *p* = 0.0011, respectively, Tukey’s test). It was also significantly higher in the US + DSS group than in the control, DSS, US, and US + DSS + VSL#3 groups (all *p* < 0.0001, Tukey’s test; Figure 2B). However, no significant group differences were revealed for neutrophil infiltration in the rectum (F = 1.217, *p* = 0.3238, one-way ANOVA; Figure 2B).

### 2.3. Gsk3β Expression in the Gut Regions

No significant differences were shown for *Gsk3β* expression in the jejunum (F = 1.348, *p* = 0.2752, one-way ANOVA; Figure 2C). *Gsk3β* expression in the ascending colon (F = 3.074, *p* = 0.031, one-way ANOVA) was significantly elevated in the US group compared to that in the DSS group (*p* = 0.0338, Tukey’s test; Figure 2C). Significant group differences were revealed for *Gsk3β* expression in the transverse colon (F = 13.38, *p* < 0.0001, one-way ANOVA). It was significantly elevated in the US group compared to the control and DSS groups (*p* = 0.0006 and *p* = 0.0018, respectively, Tukey’s test). It was also significantly higher in the US + DSS group than in the control and DSS groups (both *p* < 0.0001, Tukey’s test). Finally, it was significantly higher in the US + DSS + VSL#3 group than in the controls (*p* = 0.0359, Tukey’s test; Figure 2C). Significant group differences were revealed for *Gsk3β* expression in the descending colon (F = 23.4, *p* < 0.0001, one-way ANOVA). The US group had this measure higher than the control, DSS, and US + DSS + VSL#3 groups (*p* < 0.0001, *p* < 0.0001, and *p* = 0.0229, respectively, Tukey’s test). It was also significantly higher in the US + DSS group than in the control and DSS only groups (both *p* < 0.0001, Tukey’s test). This measure was significantly increased in the US + DSS + VSL#3 group compared to the control and DSS groups (*p* = 0.0028 and *p* = 0.0046, respectively; Tukey’s test; Figure 2C). No group differences were observed in the rectum (F = 2.407, *p* = 0.0714, one-way ANOVA; Figure 2C).

### 2.4. Neutrophil Infiltration into the Limbic Brain Regions

**Figure 3:**
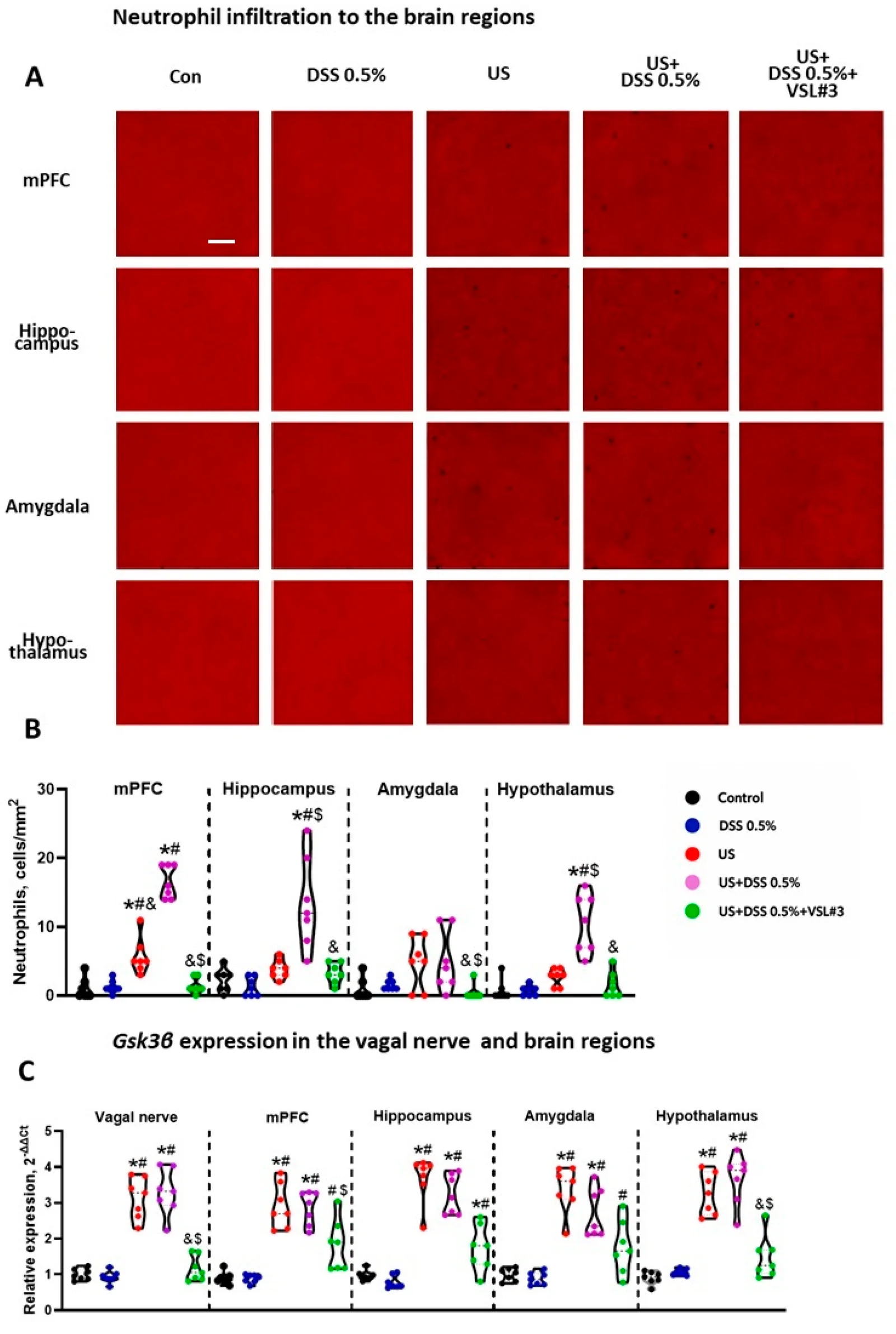
Combined exposure to US and DSS promotes neuroinflammation and *Gsk3β* upregulation in the brain. (**A**) Representative myeloperoxidase (MPO)-stained sections from the medial prefrontal cortex (mPFC), hippocampus, amygdala, and hypothalamus in the experimental groups. Dark MPO-positive cells indicate neutrophil infiltration within the limbic regions. (**B**) Quantification of MPO-positive neutrophils per mm^2^ in each brain region. (**C**) Relative *Gsk3β* mRNA expression in the vagal nerve, mPFC, hippocampus, amygdala, and hypothalamus of the brain. * *p* < 0.05 vs. control, # *p* < 0.05 vs. DSS, $ *p* < 0.05 vs. US, & *p* < 0.05 vs. US + DSS, one-way ANOVA and post hoc Tukey’s test, all n = 7. Data are shown as violin plots with individual data points. US—ultrasound, DSS—dextran sulfate sodium.

Significant group differences were found for neutrophil infiltration in the mPFC (F = 90.72, *p* < 0.0001, one-way ANOVA). It was significantly higher in the US group than in the control, DSS, and US + DSS + VSL#3 groups (*p* = 0.0004, *p* = 0.0009, and *p* = 0.0013, respectively; Tukey’s test). It was also significantly higher in the US + DSS group than in the control, DSS, US, and US + DSS + VSL#3 groups (all *p* < 0.0001, Tukey’s test; Figure 3B). The groups differed significantly in this measure in the hippocampus (F = 15.96, *p* < 0.0001, one-way ANOVA). This indicator was significantly higher in the US + DSS group than in the control, DSS, US, and US + DSS + VSL#3 groups (all *p* < 0.0001, Tukey’s test; Figure 3B). Significant group differences were found for neutrophil infiltration in the amygdala (F = 4.582, *p* = 0.0052, one-way ANOVA). The US + DSS + VSL#3 group had a significantly lower value than the US and US + DSS groups (*p* = 0.0377 and *p* = 0.03, respectively, Tukey’s test; Figure 3B). Finally, significant group differences were observed in neutrophil infiltration in the hypothalamus (F = 23.16, *p* < 0.0001, one-way ANOVA). This indicator was significantly increased in the US + DSS group compared to the control, DSS, US, and US + DSS + VSL#3 groups (all *p* < 0.0001, Tukey’s test; Figure 3B).

### 2.5. Gsk3β Expression in the Vagal Nerve and Brain Regions

Significant group differences were revealed for *Gsk3β* expression in the vagal nerve (F = 54.88, *p* < 0.0001, one-way ANOVA). It was significantly higher in the US and US + DSS groups than in the control, DSS, and US + DSS + VSL#3 groups (all *p* < 0.0001, Tukey’s test; Figure 3C). Significant group differences were shown for *Gsk3β* expression in the mPFC (F = 30.1, *p* < 0.0001, one-way ANOVA). It was significantly higher in the US group than in the control, DSS, and US + DSS + VSL#3 groups (*p* < 0.0001, *p* < 0.0001, and *p* = 0.0016, respectively, Tukey’s test). Similarly, it was significantly higher in the US + DSS group than in the control, DSS, and US + DSS + VSL#3 groups (*p* < 0.0001, *p* < 0.0001, and *p* = 0.0029, respectively; Tukey’s test). This measure was also significantly elevated in the US + DSS + VSL#3 group compared to the DSS only (*p* = 0.0084, Tukey’s test; Figure 3C). Significant group differences were observed in *Gsk3β* expression in the hippocampus (F = 50.9, *p* < 0.0001, one-way ANOVA). *Gsk3β* expression was significantly increased in the US group compared to the control, DSS, and US + DSS + VSL#3 groups (all *p* < 0.0001, Tukey’s test).

Similarly, *Gsk3β* expression was significantly higher in the US + DSS group than in the control, DSS, and US + DSS + VSL#3 groups (all *p* < 0.0001, Tukey’s test). This measure was also significantly augmented in the US + DSS + VSL#3 group compared to the controls and DSS-only group (*p* = 0.0479 and *p* = 0.0082, respectively, Tukey’s test; Figure 3C). Next, significant group differences were revealed for *Gsk3β* expression in the amygdala (F = 27.74, *p* < 0.0001, one-way ANOVA). This parameter was significantly lower in the control, DSS, and US + DSS + VSL#3 groups than in the US (all *p* < 0.0001, Tukey’s test) and US + DSS groups (*p* < 0.0001, *p* < 0.0001, and *p* = 0.0219, respectively, Tukey’s test). *Gsk3β* expression was also significantly higher in the US + DSS + VSL#3 group than in the DSS group (*p* = 0.0384, Tukey’s test; Figure 3C). Finally, significant group differences were observed in *Gsk3β* expression in the hypothalamus (F = 46.99, *p* < 0.0001, one-way ANOVA). *Gsk3β* expression was significantly elevated in the US and US + DSS groups compared to the control, DSS, and US + DSS + VSL#3 groups (all *p* < 0.0001, Tukey’s test; Figure 3C).

### 2.6. Behavioral Changes

**Figure 4:**
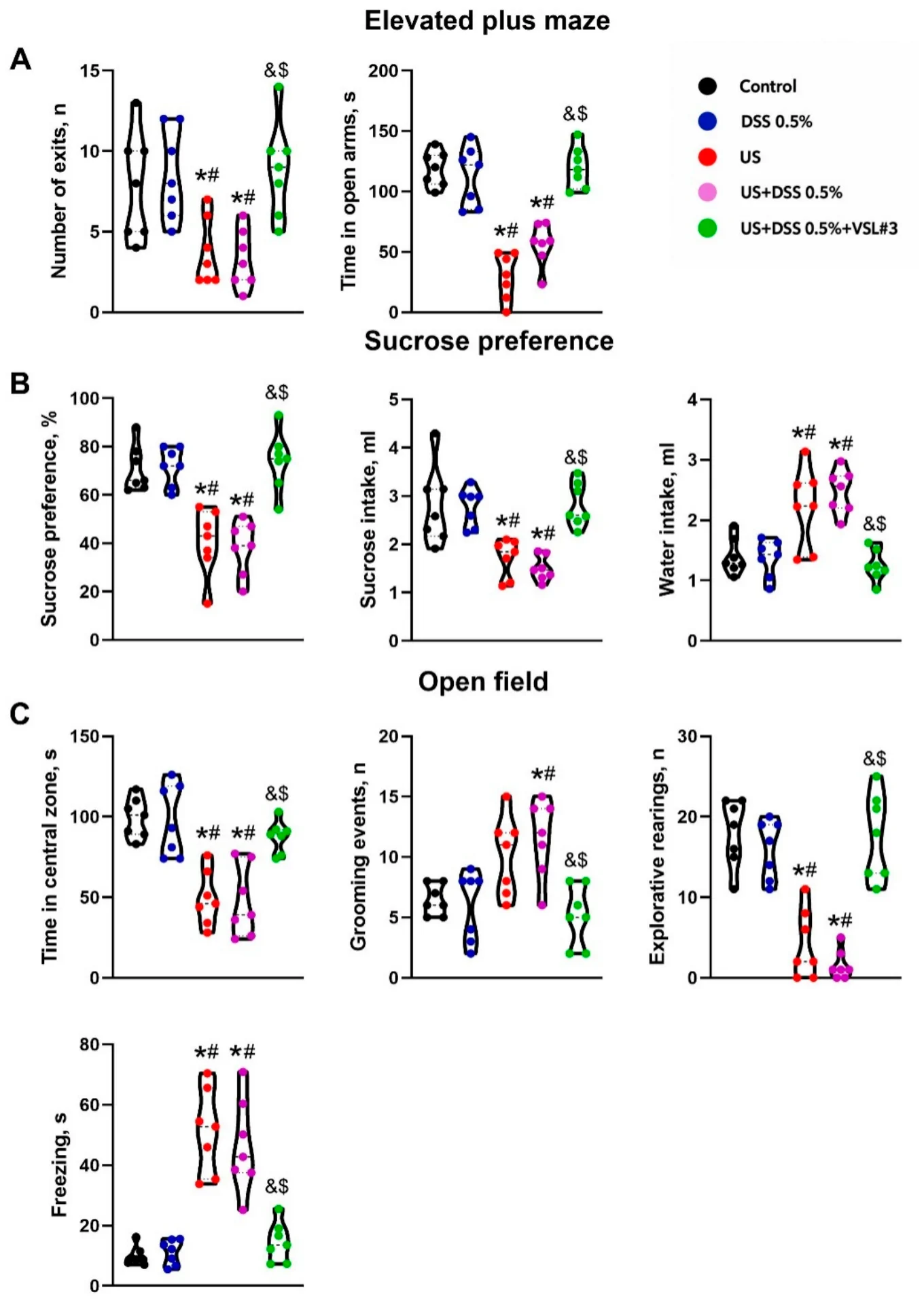
Probiotic treatment attenuates stress-induced anxiety- and depression-like behavioral scores elevated in the combined stress and DSS challenge model. (**A**) Elevated plus-maze outcomes showing the number of exits and time spent in the open arms. (**B**) Sucrose preference test outcomes showing sucrose preference, sucrose intake, and water intake. (**C**) Open field parameters showing the time spent in the central zone, grooming events, exploratory rearings, and freezing duration. * *p* < 0.05 vs. control, # *p* < 0.05 vs. DSS, $ *p* < 0.05 vs. US, & *p* < 0.05 vs. US + DSS, one-way ANOVA and post hoc Tukey’s test, all n = 7. Data are shown as violin plots with individual data points. US—ultrasound, DSS—dextran sulfate sodium.

The number of exits in the elevated plus-maze differed significantly between groups (F = 7.363, *p* = 0.0003, one-way ANOVA). This parameter was significantly higher in the control, DSS, and US + DSS + VSL#3 groups than in the US only group (*p* = 0.0486, *p* = 0.024, and *p* = 0.0089, respectively, Tukey’s test) and in the US + DSS group (*p* = 0.0174, *p* = 0.0068, and *p* = 0.04, respectively, Tukey’s test; Figure 4A). The time spent in the open arms also differed significantly (F = 34.41, *p* < 0.0001, one-way ANOVA). Similarly, this measure was significantly higher in the control, DSS, and US + DSS + VSL#3 groups than in the US only and US + DSS groups (all *p* < 0.0001, Tukey’s test; Figure 4A).

Significant group differences were revealed for sucrose preference (F = 18.66, *p* < 0.0001, one-way ANOVA). This measure was significantly higher in the control, DSS, and US + DSS + VSL#3 groups than in the US only and US + DSS groups (all *p* < 0.0001, Tukey’s test; Figure 4B). Sucrose intake also differed significantly (F = 12.07, *p* < 0.0001, one-way ANOVA). It was significantly higher in the control, DSS, and US + DSS + VSL#3 groups than in the US only group (*p* = 0.003, *p* = 0.0037, and *p* = 0.0022, respectively, Tukey’s test) and in the US + DSS group (*p* = 0.0003, *p* = 0.0004, and *p* = 0.0002, respectively, Tukey’s test; Figure 4B). Water intake also differed significantly between the groups (F = 13.7, *p* < 0.0001, one-way ANOVA). It was significantly lower in the control, DSS, and US + DSS + VSL#3 groups than in the US only group (*p* = 0.055, *p* = 0.0037, and *p* = 0.0008, respectively, Tukey’s test) and in the US + DSS group (*p* = 0.0002, *p* = 0.0001, and *p* < 0.0001, respectively; Figure 4B).

In the open field test, the time spent in the central zone differed significantly between the groups (F = 15.47, *p* < 0.0001, one-way ANOVA). This parameter was significantly elevated in control, DSS and US + DSS + VSL#3 groups compared to US only group (*p* < 0.0001, *p* < 0.0001 and *p* = 0.0024, respectively, Tukey’s test) and to US + DSS group (*p* < 0.0001, *p* < 0.0001 and *p* = 0.0014, respectively, Tukey’s test; Figure 4C). The number of grooming events also differed significantly (F = 7.568, *p* = 0.0002, one-way ANOVA). It was significantly higher in the US + DSS group than in the control, DSS, and US + DSS + VSL#3 groups (*p* = 0.0107, *p* = 0.005, and *p* = 0.001, respectively, Tukey’s test) and in the US group than in the US + DSS + VSL#3 group (*p* = 0.0137, Tukey’s test; Figure 4C). Significant group differences were revealed for the number of rearings (F = 27.03, *p* < 0.0001, one-way ANOVA). This indicator was significantly higher in the control, DSS, and US + DSS + VSL#3 groups than in the US only and US + DSS groups (all *p* < 0.0001, Tukey’s test; Figure 4C). Finally, significant group differences were shown for duration of freezing events (F = 29.11, *p* < 0.0001, one-way ANOVA). It was significantly shorter in the control, DSS, and US + DSS + VSL#3 groups than in the US only and US + DSS groups (all *p* < 0.0001, Tukey’s test; Figure 4C). Mice that received 5% DSS shown significant behavioral abnormalities in the battery of behavioral tests used (see Supplementary File).

### 2.7. Serum Inflammatory Markers

**Figure 5:**
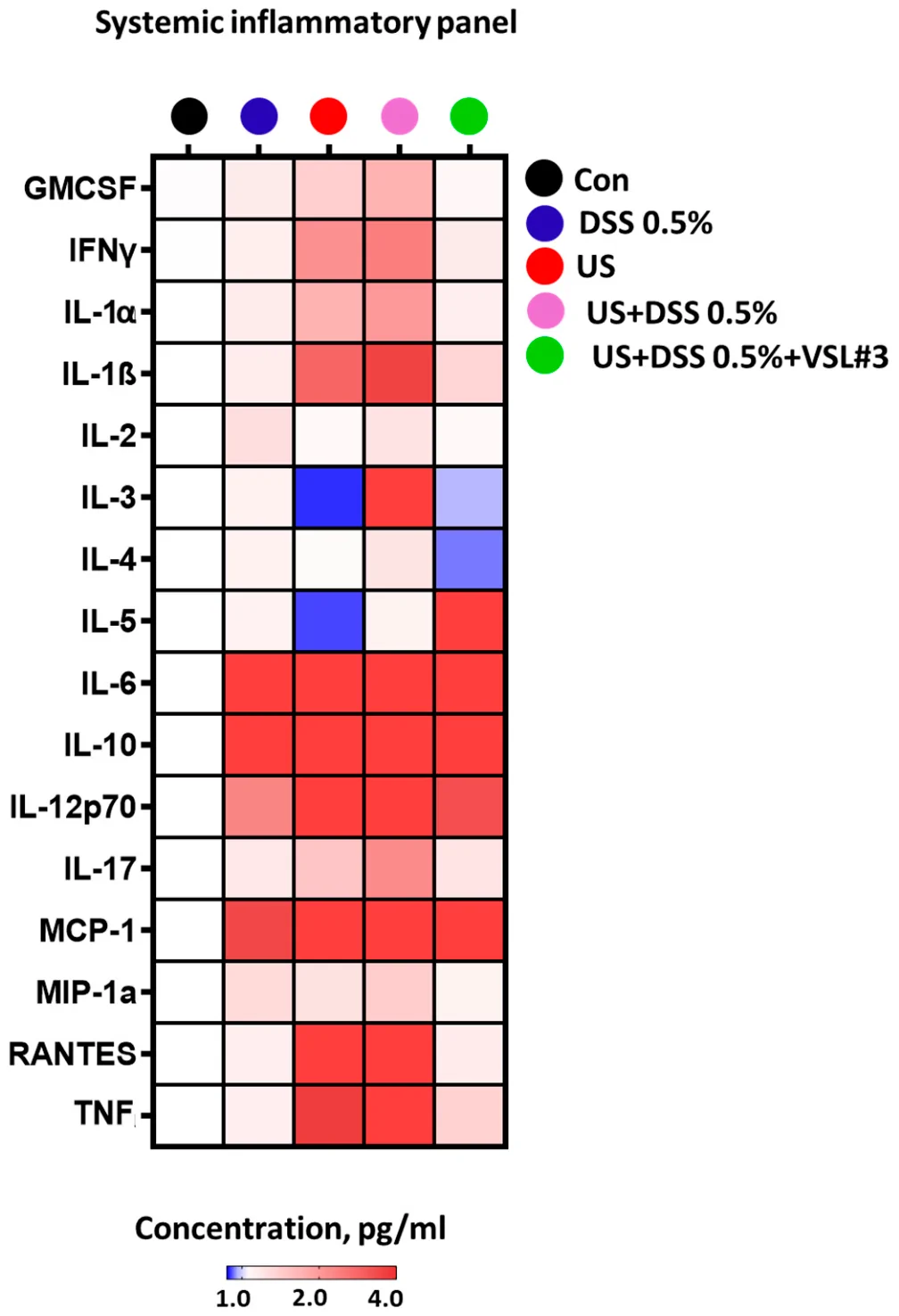
Combined stress and DSS challenge induced a serum increase in pro-inflammatory markers that was partially attenuated by probiotic treatment. Heat map of serum cytokine and chemokine concentrations in the experimental groups, expressed relative to the control group; the color scale indicates lower-to-higher relative concentrations from blue/white to red. Data were analyzed by one-way ANOVA followed by Tukey’s multiple-comparisons post hoc test. All n = 7. US—ultrasound, DSS—dextran sulfate sodium.

One-way ANOVA followed by Tukey’s multiple-comparisons post hoc test of serum inflammatory markers revealed a pronounced pro-inflammatory response in the US + DSS group compared to that in the control group. Significant group differences were found for granulocyte-macrophage colony-stimulating factor (GMCSF) concentration (F = 6.177, *p* = 0.0009, one-way ANOVA). It was significantly higher in the US + DSS group than in the control, DSS and US + DSS + VSL#3 groups (*p* = 0.0019, *p* = 0.0257 and *p* = 0.004, respectively, Tukey’s test; Figure 5). IFNγ concentration differed significantly between groups (F = 21.37, *p* < 0.0001, one-way ANOVA). Both the US and US + DSS groups had this measure significantly higher than in the control group (both *p* < 0.0001, Tukey’s test), DSS group (*p* = 0.0001 and *p* < 0.0001, respectively) and US + DSS + VSL#3 group (*p* = 0.0003 and *p* < 0.0001, respectively; Figure 5). Significant group differences were found for Il-1α concentration (F = 15.95, *p* < 0.0001, one-way ANOVA). Similarly, both the US and US + DSS groups had this indicator significantly higher than in the control group (*p* = 0.0001 and *p* < 0.0001, respectively, Tukey’s test), DSS group (*p* = 0.0069 and *p* < 0.0001, respectively) and US + DSS + VSL#3 group (*p* = 0.0046 and *p* < 0.0001, respectively; Figure 5).

IL-1β concentration differed significantly between groups as well (F = 17.58, *p* < 0.0001, one-way ANOVA). Again, it was significantly higher in the US and US + DSS groups than in the control group (both *p* < 0.0001, Tukey’s test), DSS group (*p* = 0.0004 and *p* < 0.0001, respectively) and US + DSS + VSL#3 group (*p* = 0.0042 and *p* = 0.0002, respectively; Figure 5). IL-2 concentration differed significantly (F = 6.888, *p* = 0.0005, one-way ANOVA). It was significantly higher in the DSS group than in the control, US and US + DSS + VSL#3 groups (*p* = 0.0029, *p* = 0.0164 and *p* = 0.0224, respectively, Tukey’s test). It was also significantly higher in the US + DSS group than in the control and US groups (*p* = 0.0093 and *p* = 0.0467, respectively; Figure 5). IL-3 concentration differed significantly as well (F = 10.19, *p* < 0.0001, one-way ANOVA). It was significantly higher in the US + DSS group than in the control, DSS, US and US + DSS + VSL#3 groups (*p* = 0.0012, *p* < 0.0001, *p* = 0.0083 and *p* = 0.0024, respectively, Tukey’s test; Figure 5).

Significant group differences were found for IL-4 concentration (F = 5.688, *p* = 0.0016, one-way ANOVA) which was significantly increased in the US + DSS group compared to the control, US and US + DSS + VSL#3 groups (*p* = 0.0065, *p* = 0.0295 and *p* = 0.0019, respectively, Tukey’s test; Figure 5). Next, significant group differences were found for IL-5 concentration (F = 3.871, *p* = 0.0119, one-way ANOVA); it was significantly lower in DSS and US + DSS groups compared to US + DSS + VSL#3 group (*p* = 0.046 and *p* = 0.0381, respectively, Tukey’s test; Figure 5). IL-6 concentration differed significantly as well (F = 80.17, *p* < 0.0001, one-way ANOVA). It was significantly higher in the US + DSS group compared to the control, DSS and US + DSS + VSL#3 groups (all *p* < 0.0001, Tukey’s test) and US group (*p* = 0.0013). It was also significantly higher in US group than in control, DSS and US + DSS + VSL#3 groups (all *p* < 0.0001, Tukey’s test). Finally, it was significantly higher in DSS group than in US + DSS + VSL#3 group (*p* = 0.0446; Figure 5). Significant group differences were found for IL-10 concentration (F = 17.51, *p* < 0.0001, one-way ANOVA). DSS (*p* = 0.0011, Tukey’s test), US, US + DSS and US + DSS + VSL#3 groups (all *p* < 0.0001) had this indicator significantly higher than controls; it was also significantly higher in US + DSS group than in DSS group (*p* = 0.003; Figure 5). IL-12p70 concentration differed significantly as well (F = 48.95, *p* < 0.0001, one-way ANOVA). It was significantly higher in both US and US + DSS groups than in control, DSS and US + DSS + VSL#3 groups (all *p* < 0.0001, Tukey’s test); it was also significantly higher in US + DSS group than in US group (*p* = 0.0188; Figure 5). Significant group differences were revealed for IL-17 concentration (F = 6.928, *p* = 0.0004, one-way ANOVA) which was significantly elevated in the US + DSS group compared to the control, DSS and US + DSS + VSL#3 groups (*p* = 0.0003, *p* = 0.0051 and *p* = 0.0075, respectively, Tukey’s test; Figure 5).

Monocyte chemoattractant protein-1 (MCP-1) concentration showed significant group differences (F = 26.32, *p* < 0.0001, one-way ANOVA). This measure was significantly higher in US + DSS group than in control, DSS and US + DSS + VSL#3 groups (all *p* < 0.0001, Tukey’s test) and US group (*p* = 0.0206). It was also significantly elevated in the US group compared to the control and DSS group (*p* < 0.0001 and *p* = 0.0016, respectively) and in US + DSS + VSL#3 group than in controls (*p* = 0.0158; Figure 5). Macrophage inflammatory protein-1α (MIP-1α) concentration did not differ significantly (F = 2.068, *p* = 0.11, one-way ANOVA). RANTES concentration showed significant group differences (F = 99.17, *p* < 0.0001, one-way ANOVA). This measure was significantly higher in both the US and US + DSS groups than in the control, DSS and US + DSS + VSL#3 groups (all *p* < 0.0001, Tukey’s test; Figure 5). Finally, significant differences were found for TNF concentration (F = 30.09, *p* < 0.0001, one-way ANOVA). Similarly, this measure was significantly higher in both the US and US + DSS groups than in the control (both *p* < 0.0001, Tukey’s test), DSS (*p* = 0.0002 and *p* < 0.0001, respectively) and US + DSS + VSL#3 group (*p* = 0.0018 and *p* < 0.0001, respectively). It was significantly elevated in US + DSS group than in US group (*p* = 0.0137; Figure 5).

## 3. Discussion

The present study proposes combined exposure of mice to the US and an IBS-like challenge using a drinking 0.5% DSS solution as a model of the transition from IBS- to IBD-like syndrome of profound intestinal inflammation transitions. Here, mice subjected to the combined challenge exhibited delayed intestinal transit in the rectal and whole-gut motility tests, increased fecal water content, enhanced neutrophil infiltration in the transverse and descending colon, the prefrontal cortex, hippocampus and hypothalamus, as well as elevated serum levels of pro-inflammatory mediators, particularly IL-17, TNF, MCP-1 and MIP-1α. Notably, these mice showed upregulated *Gsk3β* expression in the transverse and descending colon, as well as in the vagal nerve, prefrontal cortex, hippocampus, hypothalamus and amygdala. These changes were accompanied by anxiety-like and anhedonia-like behaviors in the elevated plus maze, open field and sucrose test. However, the combination of two stressors did not aggravate behavioral abnormalities more than the US exposure alone. Standard probiotic supplementation with VSL#3 attenuated most of the physiological, inflammatory, and behavioral changes induced by combined challenges. Together, our findings suggest that experimental ‘emotional stress’ mimicked in our study using the US model can trigger the transition from IBS-like to IBD-like pathology.

Combined exposure to ‘emotional stress’ and a low dose of DSS induced IBD-like changes that are consistent with the literature describing the effects of high doses of DSS. For example, previous studies using a 5% DSS drinking solution induced pronounced IBD-like colitis in C57BL/6 mice, characterized by progressive body weight loss, increased constipation or diarrhea, rectal bleeding, extensive epithelial damage, crypt injury, and marked infiltration of neutrophils into the colonic mucosa and muscularis externa [75,76,77,78]. Similarly to our findings, at a low dose, DSS administration was previously shown to exert only limited epithelial barrier disruption and mild inflammatory activation, without the full spectrum of clinical and histopathological features associated with overt IBD-like colitis [79,80].

Based on these findings, we interpret the combined US + 0.5% DSS paradigm as reflecting a progression from an IBS-like to an IBD-like phenotype. Low-dose DSS alone has been reported to induce colonic hypersensitivity with only mild inflammatory changes and without overt histological injury, consistent with IBS-like or remission-stage features [81,82]. In contrast, conventional DSS colitis is characterized by neutrophil infiltration, increased MPO activity, systemic and local inflammation, and mucosal injury [75,76]. In our study, 0.5% DSS administration in combination with chronic US stress resulted in marked gastrointestinal dysfunction, regional neutrophil infiltration, increased *GSK3β* expression, systemic cytokine/chemokine activation, and neuroinflammatory changes. These alterations are comparable to those observed in a parallel 5% DSS colitis model and are consistent with commonly accepted features of IBD [83,84]; see also Supplementary File.

Interestingly, our results demonstrated the specific involvement of the transverse and descending colon in the inflammatory response, which was also found in previous studies and may reflect regional differences in microbial composition, neuroimmune regulation, and luminal stasis, rendering these segments particularly vulnerable to neutrophil infiltration [85]. The selective upregulation of *Gsk3β* in the transverse and descending colon, but not in the jejunum, ascending colon, or rectum, was found in the group exposed to the combined challenge as well as to the US alone. This regional susceptibility corresponds well with the neutrophil infiltration data and may additionally highlight differences in enteric neural organization, microbial composition, epithelial barrier function, and immune surveillance along the gastrointestinal tract of mice.

Several mechanisms may underlie this regional pattern. DSS directly disrupts the intestinal mucosa and epithelial barrier, so differences in luminal exposure, transit time, mucus organization, and epithelial susceptibility may shape local inflammation [75,86]. In our study, slowed transit and reduced pellet output may have prolonged mucosal DSS exposure, while progressive reduction in DSS concentration toward distal segments could contribute to the absence of significant rectal inflammation. Regional differences in stress-sensitive barrier function may also be involved [64]. In addition, proximal, middle, and distal colonic segments differ in vagal and sympathetic innervation [87,88,89], and adrenergic signaling regulates neutrophil recruitment [63]. A lack of significant neutrophil infiltration of the rectum, whose motor functions were yet affected in challenged animals, can be explained by the shorter exposure of this part of the colon to luminal content and lower DSS concentration at the distal parts of the gut.

Consistent with this interpretation, GSK3β is a key regulator of inflammatory signaling, epithelial barrier dysfunction, and neuroimmune activation, and its dysregulation has been implicated in intestinal inflammatory disorders [26,90]. Rather than indicating direct activation of inflammation by stress alone, altered *Gsk3β* expression may reflect a molecular state in which immune and stress-responsive signaling networks become more readily activated following an additional inflammatory challenge. This interpretation is consistent with the established role of *Gsk3β* as a regulatory node integrating inflammatory pathways, including NF-κB-dependent cytokine production, neutrophil activation, and cellular responses to stress [91,92,93].

The partial normalization of IBD-like changes in mice that received VSL#3 probiotic supplementation suggests that stress-induced colitis may involve microbiota-dependent regulation of inflammatory and stress-responsive pathways [94,95]. Such effects may occur through several mechanisms, including restoration of intestinal barrier function, modulation of microbial metabolite production, and regulation of immune–neural communication [96]. For example, microbiota-derived metabolites, particularly short-chain fatty acids such as butyrate, can influence epithelial integrity, immune cell differentiation, and inflammatory signaling, thereby promoting maintenance of intestinal immune homeostasis [97]. In addition, VSL#3 probiotic can enhance epithelial barrier function by regulating tight junction integrity [98]. Microbial short-chain fatty acids can also alter innate immune responses through pattern-recognition receptors, which regulate downstream NF-κB activation and cytokine production of IL-1 family [99]. Studies on Wistar and Sprague-Dawley rats have demonstrated the efficacy of probiotic supplementation VSL#3 on normalizing NO synthase and NADPH oxidase subunits expression, as well as preservation hippocampal neuroplasticity by upregulation of BDNF level [100,101]. Together, the ability of probiotic supplementation VSL#3 to reverse stress-associated molecular and behavioral alterations suggests validate the paradigm of combined US and DSS challenge as a model of a transition of IBS to IBD-like state.

Interestingly, probiotic supplementation counteracted the negative effects on behavior in US-exposed mice. This can be explained by the normalization of stress-induced oxidative and inflammatory changes by the administration of VSL#3. Previously, these changes and behavioral abnormalities in the US models were shown to be reversible by anti-inflammatory and anti-oxidant agents thiamine and benfotiamine, which also attenuated oxidative stress and restored plasticity-associated protein expression, supporting a mechanistic link between GSK3β-associated signaling and oxidative stress regulation in the model of ‘emotional stress’ [36,37]. In addition, in the prefrontal cortex and hippocampus, neuroinflammation and oxidative stress markers were previously shown to positively correlate with GSK3β upregulation [23,37].

Remarkably, this study showed the upregulation of *Gsk3β* gene expression in the vagal nerve in both the US and double-challenge groups. This finding is relevant to the neuroimmune interpretation of our model because the nervus vagus serves as a major bidirectional communication pathway between the gastrointestinal tract and the brain, contributing to both the afferent and efferent regulation of intestinal immune activity [102,103]. Vagal afferent fibers respond to peripheral inflammatory cytokines, such as IL-1β and TNF, as well as gastrointestinal distension, and activate the brainstem, particularly the nucleus tractus solitarius resulting in central integration of gastrointestinal and immune status via parabrachial nuclei of amygdala [103,104,105]. Vagal efferent signaling can regulate peripheral inflammation through the cholinergic anti-inflammatory pathway, in which acetylcholine acts primarily through α7 nicotinic acetylcholine receptors on immune cells to suppress the production of pro-inflammatory mediators, including TNF, IL-1β, and IL-6 [102,103,106].

Gene expression analysis showed that in the US-DSS model, the gut, nervus vagus, and brain displayed upregulated GSK3β, which is known to be suppressed by acetylcholine acting on α7 nicotinic receptors through the JAK2–PI3K/Akt and NF-κB cascades [107]. This suggests diminished cholinergic signaling in the employed model; the changes observed in the vagal tissue do not suggest increased firing or acetylcholine release, which is in line with previous observations [108]. In contrast, elevated *Gsk3β* mRNA accompanied by proinflammatory changes points to a decrease in parasympathetic tone. In parallel, stress-related sympathetic activation and noradrenaline release [65] are likely to promote neutrophil recruitment in DSS colitis through adrenergic receptors [63] and activate microglia in the hippocampus and hypothalamus via β-adrenergic signalling [109].

As shown in previous observations, ‘psychological’ or ‘emotional’ stress in rodents alone does not produce significant pathological colitis-like changes in the gut [20,21]. However, some of the alterations reported in our work were similar to those observed in the group exposed to the combined challenge. Namely, we found that US-exposed mice displayed significant upregulation of *Gsk3β* in the vagal nerve and in all investigated brain areas, as well as in the transverse and descending gut sections. In these mice, neutrophil infiltration was shown in the prefrontal cortex, hippocampus, and above-mentioned gut regions. These pathological changes may underpin the mechanisms of triggering a transition from the IRS to the IRD state and be associated with previously demonstrated systemic inflammation and oxidative stress in this paradigm [36,37,110].

Another important mechanism can be the alteration of the intestinal microbiota [111,112]. In line with the results reported here, chronic unpredictable stress has been shown to promote colonic neutrophil infiltration that was mediated by increases in adrenergic signaling, expression of pro-inflammatory cytokines, and neutrophil-recruiting chemokines [63]. Additionally, chronic unpredictable mild stress has been shown to alter gut microbial composition, impair epithelial barrier integrity, and amplify mucosal immune responses, increasing the susceptibility of the colon to inflammatory insults [111]. Furthermore, foot shock stress was found to exacerbate IL-18-dependent enhancement of TNF production, followed by a 5-day exposure to a 3% DSS administration [113].

Stress-induced signs of colitis reported here in the US group of mice are in line with clinical observations. Prospective cohort studies have shown that psychological stress, including long-term anxiety, frustration and depression-related symptoms, can worsen motility to both constipation or diarrhea, stool loosening, and increase relapse risk in both IBS and IBD [4,18,114,115]. In particular, in a longitudinal 16 months study of 117 IBS patients, chronic life stress scores were reported to be related to colitis severity. It was demonstrated that chronic life stress was a strong predictor of subsequent IBS symptom intensity, indicating that persistent stress exposure contributes to gastrointestinal symptom severity rather than merely reflecting the presence of symptoms [116]. Similarly, in a one-year follow-up study, Whitehead et al. showed that in 383 women with or without IBS symptoms, stressful life events were closely associated with reported gastrointestinal problems [117]. Recent studies have yielded similar outcomes [118,119,120,121,122,123] and additionally to above-mentioned pre-clinical studies demonstrated that the alteration of the intestinal microbiota can contribute to aggravation of colitis [111,112]. For example, in patients with ulcerative colitis, altered abundance of *Enterococcus spp*., *Pseudomonas spp*., *E. coli biovare*, and *Candida albicans*, have been reported alongside elevated markers of intestinal permeability and inflammation [112].

Here we also showed that mice exposed to a combined challenge of US and DSS displayed a sustained increase in circulating inflammatory mediators, including IL-1 family cytokines, IL-6, IL-12p70, IL-17, MCP-1, MIP-1α, TNF, and IFNγ. Rather than representing a physiological adaptive response, this systemic cytokine upregulation profile is consistent with a dysregulated inflammatory state characterized by persistent immune activation contributing to colitis [124]. Excessive sustained production of pro-inflammatory mediators can disrupt immune balance in a gut, promoting leukocyte recruitment, epithelial dysfunction, and impaired tissue recovery [125,126]. This observation is consistent with clinical and experimental evidence demonstrating that excessive activation of IL-1β, TNF, IL-6, IL-17, and chemokine networks contributes to intestinal immune amplification in patients with IBD [124,127]. Notably, ‘emotional’ stress alone and exposure to stressors inducing the MDD-like state and anxiety-like syndrome in rodents are shown to cause ‘sterile’ inflammation alone [35,128]. This is in keeping with clinical observations in patients with MDD, where peripheral IL-6 and CRP are consistently elevated, and with reports of comorbid anxiety [129,130]. Therefore, the circulating inflammatory signature observed following the challenge may represent aan additional mechanistic link between psychological stress, enhanced immune activation, and the development of IBD-like syndrome in our study.

In summary, our work demonstrates that experimental ‘emotional stress’ induced in mice by the US can trigger a transition from IBS to IBD syndrome. The sensitivity of this pathological development to standardized validated anti-inflammatory probiotic treatment validates this paradigm as an inflammation-based model and points to the importance of gut microbiota in the transition from IBS to IBD. Our study demonstrated the role of GSK3β pathway activation and the involvement of the vagal nerve in disease progression from IBS to IBD. At the same time, a lack of direct morphological evidence of IBD syndrome and the relatively small group size should be taken into account when interpreting the negative findings, although the reported group differences were robust and consistent, and histological changes corresponding to the experimental gut pathology described here are well established.

## 4. Materials and Methods

### 4.1. Animals

Male C57BL/6J mice (5–6 months of age) were obtained from a certified Charles River provider (Stolbovaja, RAS, http://www.spf-animals.ru/about/providers/animals (accessed on 2 February 2023)). Animals were housed individually in standard plastic cages and maintained on a reverse 12 h light/dark cycle (lights on at 19:00) under controlled laboratory conditions (22 ± 1 °C, ~55% humidity), with food and water provided ad libitum unless otherwise required for gut motility testing. All behavioral experiments were conducted during the dark phase. Animals were monitored daily in the morning and evening to assess general health and to minimize potential confounding factors. All housing conditions and experimental procedures complied with the ARRIVE guidelines and Directive 2010/63/EU (22 September 2010) and were approved by the local veterinary committee (accepted on 24 May 2019, N101). All efforts were made to minimize animal discomfort. The study did not include predefined humane endpoints, and no animals were excluded from the study.

### 4.2. Study Flow

**Figure 6:**
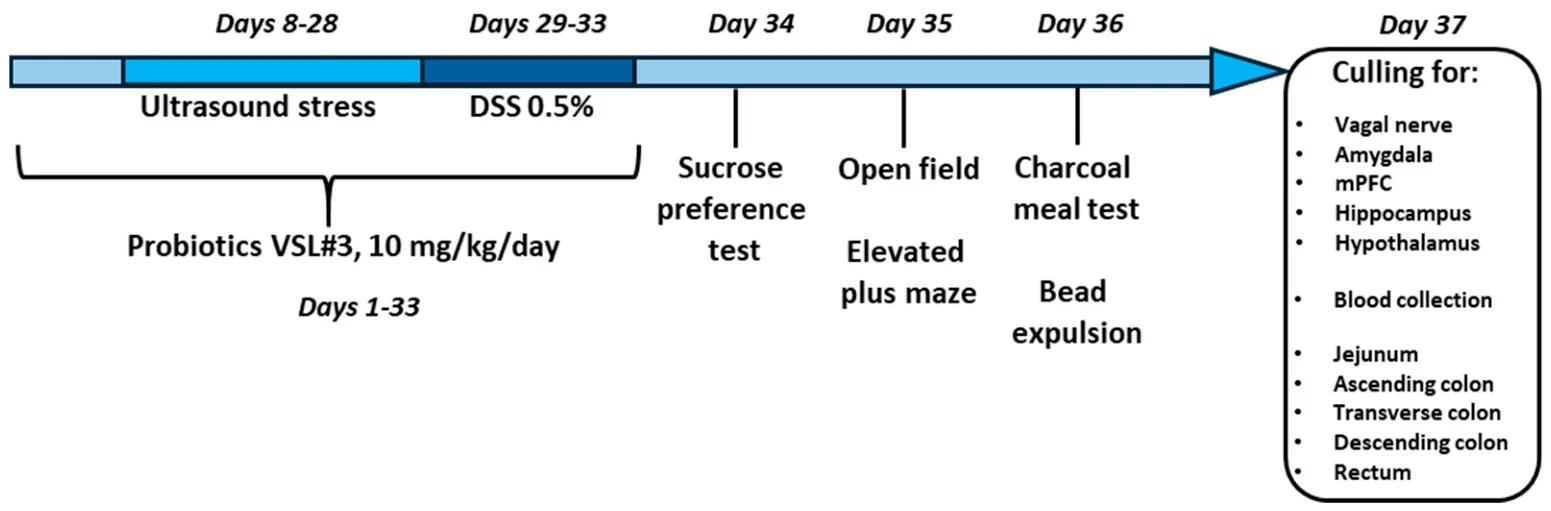
Experimental design. Groups of mice received probiotics or vehicle for 33 days (days 1–33), starting 7 days before the onset of stress and continuing throughout stress and DSS exposure. Ultrasound stress was applied on days 8–28, followed by DSS 0.5% in the drinking water on days 29–33. On day 34, mice underwent sucrose preference test. On day 35, open field and elevated plus maze tests were conducted. On day 36, charcoal meal tests and bead expulsion tests were performed. On day 37, mice were euthanized, and their blood, brain, and intestines were collected. DSS—dextran sulfate sodium, mPFC—medial prefrontal cortex.

Mice were assigned to five experimental groups: (1) control non-stressed, non-treated mice (n = 7); (2) non-stressed mice receiving DSS (0.5%) (n = 7); (3) mice exposed to US (n = 7); (4) mice subjected to combined ultrasound stress and DSS (0.5%) as a combined challenge paradigm (n = 7); and (5) mice subjected to the combined challenge and treated with the probiotic formulation VSL#3 (n = 7). A separate DSS-control group of male C57BL/6 mice received 5% DSS in the drinking water for 5 days (n = 7); mice were studied for behavior and the charcoal meal and bead expulsion tests (see below). In total, 42 mice were used. Sample sizes were selected based on previous studies and adjusted to balance statistical power with ethical considerations. Randomization was performed with stratification based on body weight and age to ensure balanced group allocation. Probiotic or vehicle treatment was administered daily for 33 consecutive days (days 1–33), beginning 7 days before the onset of stress and continuing throughout stress and DSS exposure. For the combined challenge paradigm, mice were exposed to ultrasound stress for 3 weeks (days 8–28), after which DSS (0.5%) was administered in the drinking water for 5 consecutive days (days 29–33), without overlap with the stress period. Behavioral testing was conducted in the following sequence: sucrose preference test, open field test, elevated plus maze test, charcoal meal test, and bead expulsion test, on days 34–36 (see Figure 6). Mice were then terminally anesthetized with isoflurane, and blood samples were collected via transcardial puncture for assessment of systemic inflammatory markers as described elsewhere [36,37,57,58,59,75]. Animals were subsequently perfused with phosphate-buffered saline (PBS, pH 7.4), and the brain, nervus vagus and gastrointestinal tissues were harvested for analysis of immune cell infiltration and molecular assays, including assessment of *Gsk3β* expression (see below). All behavioral, gastrointestinal, and molecular analyses were performed by investigators blinded to group allocation. Data processing and statistical analysis were also conducted under blinded conditions.

### 4.3. Ultrasound Stress

The ultrasound stress procedure was performed as described previously [14,37]. Emotional stress was induced by continuous 21-day exposure to unpredictable alternating ultrasonic frequencies delivered in the animal housing room using a commercial device (Weitech, Wavre, Belgium). Ultrasound stimulation was applied at an average intensity of 50 ± 5 dB with randomized variation within the 20–45 kHz range. The signal was delivered in a random schedule of alternating 10 min intervals across low (20–25 kHz), middle (>25–40 kHz), and high (40–45 kHz) frequency bands with dynamic short-term fluctuations within each interval. The loudness of the sound was fluctuated within the range ± 10% of the average value, i.e., ±5 dB. The stimulation frequencies alternated at variable short time spans of ≤1 s (averaged frequency 70 ± 10 Hz) at the above-indicated intervals. Low and middle frequency ultrasound radiation constituted 35% of the emission time while high frequencies constituted 30% of the emission time. An example of US stimulation signal is presented in the Supplementary File (Figure S1). The even distribution of ultrasound radiation was controlled using an ultrasound detector (Discovery Channel, USA) for the course of the study. The ultrasound device was hung 2 m above the cages of the experimental groups with an average horizontal distance to cages of 2.5 m. The position of the cages, with respect to the stimulator, was changed weekly.

The selectivity of the adverse effects of low frequency ultrasound, during the employed radiation, versus the potential general negative effects of constant acoustic noise, was evaluated in separate experiments that were reported elsewhere [14]. In a previous study, a comparison of a 3-week exposure to 50 dB ultrasound of mixed frequencies (‘white noise’) at an average frequency of 16 kHz with the conditions used in the current experiments revealed a lack of stress-induced behavioral and other changes in the former group [14]. In a comparison of the potential immediate effects of ultrasound at ‘emotionally negative’ frequencies of 20–25 kHz and ‘emotionally neutral’ frequencies of 16 and 50–60 kHz, only the former range of frequencies caused significant behavioral effects on exploration, freezing scores, defensive burying and approaches to palatable diet manifesting stress response [14]. This stress-related effects in US paradigm employed here cannot be explained by acoustic exposure or loudness alone. The above-described US exposure parameters and behavioral and molecular readouts investigated in current study were selected based on previously validated protocols for ultrasound-induced emotional stress [14,37].

### 4.4. Induction of Intestinal Inflammation

Intestinal inflammation was induced using DSS (molecular weight 36–50 kDa; MP Biomedicals, Solon, OH, USA) administered in drinking water (see below) for 5 consecutive days, as previously described [131]. A subclinical dose of DSS at 0.5% was chosen [82]. Control mice received regular tap water. Liquid intake was monitored daily by measuring 24 h consumption.

### 4.5. Probiotics Administration

Mice received VSL#3 High Potency Probiotic Medical Food (Ferring Pharmaceuticals, Saint-Prex, Switzerland) by oral gavage using a flexible gavage needle for 33 consecutive days (days 1–33), beginning 7 days before the onset of ultrasound stress and continuing throughout stress and DSS exposure. The probiotic was dissolved in sterile water at 4 × 10^8^ CFU/mL and delivered in a total volume of 0.5 mL, twice a day, 8 h apart, as described elsewhere [132]. VSL#3 is a commercially available standardized multi-strain formulation containing freeze–dried *Bifidobacterium longum*, *B. breve*, *B. infantis*, *Lactobacillus acidophilus*, *L. plantarum*, *L. casei*, *L. helveticus*, and *Streptococcus thermophilus* [68,73]. Control animals received an equivalent volume of sterile saline by oral gavage following the same schedule.

### 4.6. Behavioral Tests

#### 4.6.1. Sucrose Test

Mice were given a 24 h free-choice between a bottle containing 1% sucrose solution and a second bottle containing tap water, as previously described [23,59]. To avoid side bias, bottle positions were switched halfway through the testing period. Fluid consumption was determined by weighing bottles at the start and end of the test. Sucrose preference was calculated as follows: Sucrose preference (%) = [volume of sucrose/(volume of sucrose + volume of water)] × 100% [23,59].

#### 4.6.2. Open Field Test

The open field test was conducted in square arenas (45 × 45 × 45 cm) made of gray plastic (Technosmart, Rome, Italy) and illuminated under white light (25 lux) [57,133]. Mice were gently placed near the arena wall, and exploratory behavior was recorded for 5 min. The arena was cleaned between animals. The time spent in the central zone (15 × 15 cm) and the duration of freezing were quantified using automated video-tracking software (AnyMaze Version 7.68; Stoelting Co., Wood Dale, IL, USA). The number of grooming events and the number of rearings were scored manually from the video recordings by an experimenter blinded to group allocation [57,133].

#### 4.6.3. Elevated Plus Maze

The elevated plus maze apparatus (Technosmart, Rome, Italy) was constructed from gray plastic and consisted of two open arms (25 × 5 cm, with a 0.5 cm rim) and two closed arms (25 × 5 × 16 cm) arranged perpendicularly around a central platform (5 × 5 cm), elevated 70 cm above the floor. Illumination was set at 25 lux on the central platform and open arms [57,133,134]. Mice were placed on the central platform facing an open arm and allowed to explore for 5 min. The apparatus was cleaned between animals. The number of entries into the open arms and the time spent in the open arms were recorded using automated video-tracking software (AnyMaze; Stoelting Co., Wood Dale, IL, USA) [57,134].

### 4.7. Tests for Gastrointestinal Functions

#### 4.7.1. Charcoal Meal Test

Before testing, mice were fasted for 12 h with free access to water to ensure an empty gastrointestinal tract and consistent baseline conditions. On the day of testing, each mouse received an oral gavage of 0.2 mL of a freshly prepared activated charcoal suspension using a flexible gavage needle. Following gavage, mice were housed individually in clean cages without bedding material to allow easy detection of fecal output. The animals were continuously monitored, and the time interval from gavage to the appearance of the first black-colored fecal pellet was recorded as the total gastrointestinal transit time in minutes [135]. All pellets voided were counted, and the frequency of evacuation was expressed per hour. Individual fecal pellets were weighed immediately after collection on an analytical balance to determine their wet weight, then dried in an oven at 60 °C for 24 h to obtain the dry weight; the subtraction of the dry weight from the wet weight represented fecal water [60].

#### 4.7.2. Bead Expulsion Test

Mice were briefly anesthetized with isoflurane, and a 2 mm glass bead mounted on a lubricated plunger was inserted 2 cm into the distal colon, adapted from previously described procedures [61,62]. The animals were then placed individually in a recovery chamber and monitored until full recovery from anesthesia. Bead expulsion time was defined as the interval between the recovery of spontaneous movement and the complete expulsion of the bead from the rectum [61,62].

### 4.8. Dissection of Vagus Nerve

The cervical vagus nerve was identified within the carotid neurovascular bundle (carotid sheath) and isolated as described previously [136,137]. Briefly, the submandibular/salivary glands and overlying connective tissues were gently retracted to expose the tracheal region and underlying cervical neurovascular structures. The vagus nerve was identified within the carotid sheath by its slender appearance and location adjacent to the carotid artery. Under magnification, the surrounding connective tissue was carefully dissected to visualize the nerve, which was then gently separated from the adjacent carotid artery and connective tissues while preserving its structural integrity. The isolated vagal tissue was immediately frozen and stored at −80 C° until subsequent analysis.

### 4.9. Quansys Q-PlexTM ELISA Array

Serum cytokine concentrations were measured using a mouse 16-plex Q-Plex ELISA array (Quansys Biosciences, West Logan, UT, USA) detecting GM-CSF, IFN-γ, IL-1α, IL-1β, IL-2, IL-3, IL-4, IL-5, IL-6, IL-10, IL-12p70, IL-17, MCP-1, MIP-1α, regulated upon activation, normal T-cell expressed and secreted (RANTES), and TNF [35,138]. Blood samples were centrifuged at 1500× *g* for 10 min at 4 °C, and serum was processed according to the manufacturer’s instructions. A volume of 30 µL per well was used. Signal detection was performed using a Q-View Imager (Quansys Biosciences, West Logan, UT, USA) based on a chemiluminescent readout. Cytokine concentrations were quantified using Q-View software and expressed relative to the control group [35,139].

### 4.10. Neutrophil Infiltration Analysis

Neutrophil infiltration in the intestinal and brain regions was assessed using myeloperoxidase (MPO) staining, as previously described [140,141]. Briefly, Hanker–Yates reagent (Polyscience Europe, Eppelheim, Germany) was freshly dissolved in Krebs buffer, followed by the addition of 3% H_2_O_2_, and intestinal and brain tissue preparations were incubated for MPO staining in this reaction mixture for 10 min at room temperature. The composition of the Krebs buffer used in these experiments was as follows (in mM): NaCl 120, KCl 6.0, MgCl_2_ 1.2, NaH_2_PO_4_ 1.35, NaHCO_3_ 14.4, CaCl_2_ 2.5, and glucose 12.7 (Thermo Fisher Scientific, Waltham, MA, USA). Subsequently, the tissues were rinsed extensively with Krebs buffer (3 × 10 min) before analysis [140]. The preparations were then examined under a light microscope (Olympus BX51 microscope, Olympus, Tokyo, Japan). MPO-positive cells were identified by their characteristic reaction product and quantified across randomly selected microscopic fields. Specifically, for quantitative analysis, five randomly selected, non-overlapping microscopic fields of view under a 20 × objective were examined for each sample tissue preparation, as described elsewhere [141]. The number of MPO-positive cells was counted in each field, values were averaged for each mouse used, and MPO-positive cell density was expressed as cells/mm^2^.

### 4.11. RNA Extraction and Quantitative PCR

Quantitative real-time PCR (qRT-PCR) was performed as previously described [23,57,138]. Total RNA was extracted from tissue samples using the RNeasy Lipid Tissue Mini Kit (Qiagen, Hilden, Germany). First-strand cDNA was synthesized from 1 μg of total RNA using the QuantiTect Reverse Transcription Kit (Qiagen, Hilden, Germany) following established protocols [14,23,35,57,138]. qRT-PCR was performed using a SYBR Green master mix (Bio-Rad, Hercules, CA, USA) on a CFX96 Deep Well Real-Time PCR Detection System (Bio-Rad, Hercules, CA, USA). Each 10 μL reaction contained 5 μL SYBR Green master mix, 3 μL RNase-free water, 1 μL of a forward and reverse primer mix, and 1 μL cDNA. Thermal cycling conditions consisted of an initial denaturation at 95 °C for 4 min, followed by 40 cycles of 95 °C for 20 s and 54 C for 90 s. *Gsk3β* was amplified using the primers 5′-CGAGCATGTGGAGGGATAAG-3′ (forward) and 5′-GCACTCTTCAACTTTACCACTCAAG-3′ (reverse). Glyceraldehyde-3-phosphate dehydrogenase (*Gapdh*; forward 5′-CACTGAGCATCTCCCTCACA-3′, reverse 5′-GTGGGTGCAGCGAACTTTAT-3′) was used as the reference gene because of its stable expression under experimental stress conditions [14,35,57]. All reactions were performed in triplicate. Relative gene expression was calculated using the ΔΔCt method and normalized to Gapdh, with values expressed as fold change versus control the group [22,57,138].

### 4.12. Preparation of DSS Drinking Solution

A 0.5% (*w*/*v*) DSS solution was prepared by dissolving 0.5 g of DSS powder (molecular weight 36–50 kDa; MP Biomedicals, CA, USA) in approximately 90 mL of drinking water as described elsewhere [75,76,142]. The volume was then adjusted to 100 mL. DSS was dissolved to a final concentration of 0.5% (*w*/*v*) and provided ad libitum. Fresh solution was prepared daily; special drinking bottles minimizing liquid spillage were employed [56].

### 4.13. Statistical Analysis

Statistical analyses were performed using GraphPad Prism version 8.01 (GraphPad Software, San Diego, CA, USA). Data distribution was assessed using the Shapiro–Wilk normality test. As all datasets followed a normal distribution, comparisons among multiple groups were performed using one-way analysis of variance (ANOVA) followed by Tukey’s post hoc test. Statistical significance was set at *p* < 0.05. No predefined inclusion or exclusion criteria were applied, and no data points were excluded from the analysis. Results are presented as violin plots with individual data points.

## Data Availability

The original contributions presented in this study are included in the article/supplementary material. Further inquiries can be directed to the corresponding author(s).

## Supplementary Materials

The following supporting information can be downloaded at: https://www.mdpi.com/article/10.3390/ijms27209092/s1. References in File [143, [144.

## Abbreviations

IBS	Irritable Bowel Syndrome
IBD	Inflammatory Bowel Disease
DSS	Dextran Sulfate Sodium
GSK3β	Glycogen Synthase Kinase 3 Beta
CUMS	Chronic Unpredictable Mild Stress
CpG-ODN	Cytosine–phosphate–guanine Oligodeoxynucleotide
NF-κB	Nuclear Factor kappa-light-chain-enhancer of activated B cells
CREB	cAMP Response Element-Binding Protein
IL	Interleukin
TNF	Tumor Necrosis Factor
IFNγ	Interferon gamma
CD11b^+^	Cluster of Differentiation 11b positive cells
Ly6G^+^	Lymphocyte Antigen 6 Complex, locus G positive cells
MPO	Myeloperoxidase
CXCL1	C-X-C Motif Chemokine Ligand 1
CCL2	C-C Motif Chemokine Ligand 2
TNBS	Trinitrobenzene Sulfonic acid
HPA	Hypothalamic–Pituitary–Adrenal
MAPK	Mitogen-Activated Protein Kinase
mPFC	medial Prefrontal Cortex
ANOVA	Analysis of Variance
GM-CSF	Granulocyte-Macrophage Colony-Stimulating Factor
MCP-1	Monocyte Chemoattractant Protein-1
MIP-1α	Macrophage Inflammatory Protein-1 alpha
qRT-PCR	quantitative Real-Time Polymerase Chain Reaction
RNA	Ribonucleic Acid
cDNA	complementary DNA
Gapdh	Glyceraldehyde-3-Phosphate Dehydrogenase
RANTES	Regulated upon Activation, Normal T cell Expressed and Secreted
