# Supplementary material for: Chronic Ultrasound (‘Emotional’) Stress in Mice Exacerbates Colitis Through GSK3β-Mediated Neuroimmune Dysregulation and Is Ameliorated by Probiotic Intervention
Source: Int J Mol Sci. 2026 Oct 14;27(20):9092. doi: 10.3390/ijms27209092 (PMC13704906; doi:10.3390/ijms27209092)
Supplement: Supplementary file 1 [file ijms-27-09092-s001.zip › ijms-4610095-supplementary.pdf]

Supplementary file

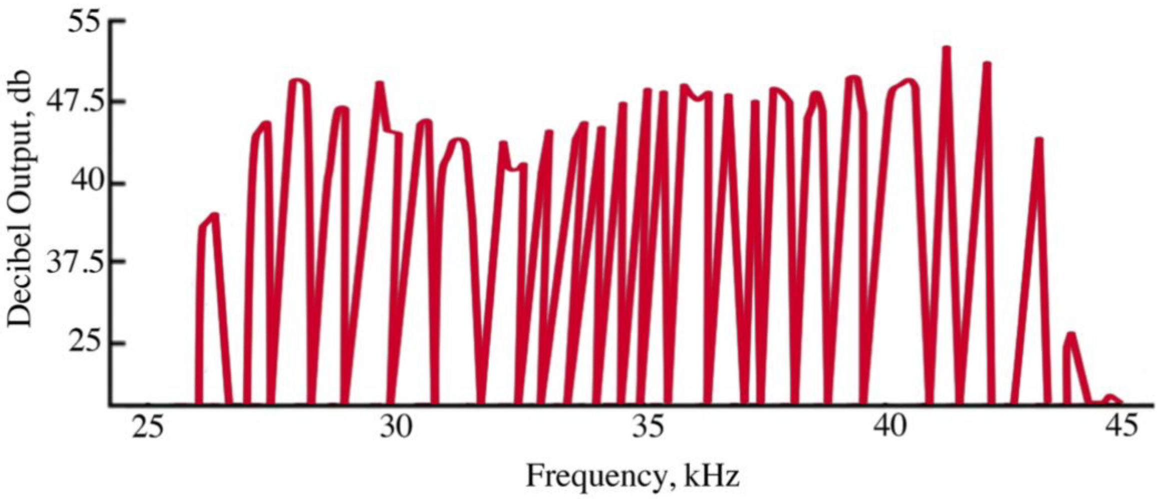

Supplementary Figure S 1. Representation of the ultrasound signal: frequency–intensity fluctuations mimicking the natural ultrasonic vocalizations of mice. During each 10-min period, ultrasound frequencies fluctuated over short time spans of ≤1 s within the corresponding interval. Low (20–25 kHz) and middle (>25–40 kHz) frequencies each accounted for 35% of the emission time, and high frequencies (40–45 kHz) for the remaining 30%. Signal intensity fluctuated within ±10% of the mean value, that is ±5 dB.

Effects of 5% DSS challenge on physiological outcomes and gut histology

A separate cohort of male C57BL/6 mice received 5% DSS (36–50 kDa; MP Biomedicals, Irvine, CA, USA) in the drinking water for 5 consecutive days, while control animals received regular tap water (n=7 per group). Following the end of DSS administration, mice underwent the sucrose preference test, the open field and elevated plus maze tests, and the charcoal meal and bead expulsion tests.

It was shown than mice exposed to 5% DDS had significantly prolonged latency to first fecal pellet evacuation (p=0.0009, t-test; Suppl. Fig. 1A) and significantly higher fecal water content (p=0.001, t-test; Suppl. Fig. 1A) in the charcoal meal test, as well as significantly increased latency to bead expulsion (p=0.0004, t-test; Suppl. Fig. 1B).

In addition, histological examination of gut tissue was performed (data not shown; Pavlov et al., in preparation). Briefly, the descending colon tissues were fixed in 4% formalin for 24 h, processed through graded ethanol and xylene, embedded in paraffin, sectioned, and stained with hematoxylin and eosin as previously described (Feldman and Wolfe, 2014). Histomorphological injury was scored according to Erben et al. (2014), and the control and 5% DSS groups were compared using an unpaired two-tailed Student's t-test ($p < 0.05$). In the 5% DSS-treated group, the normally continuous epithelial surface was disrupted, with focal epithelial attenuation and mucosal erosion, accompanied by marked goblet cell depletion. The colonic crypt architecture was also disturbed, with shortened, irregular, distorted, and partially disrupted crypts, including areas of crypt loss. Increased inflammatory cell accumulation was evident in the lamina propria and deeper tissue compartments, together with submucosal expansion consistent with edema (data not shown; Pavlov et al., in preparation). Overall, 5% DSS exposure produced characteristic features of severe DSS-associated mucosal injury, including epithelial damage, goblet cell depletion, crypt disruption, inflammation, and edema (data not shown; Pavlov et al., in preparation). These data with a 5% DSS challenge are consistent with the literature (Wirtz et al., 2007; Chassaing et al., 2014; Bessac et al., 2019). Functional and inflammatory changes in 5%DSS-exposed mice were comparable to those described in the US+0.5% DSS mice.

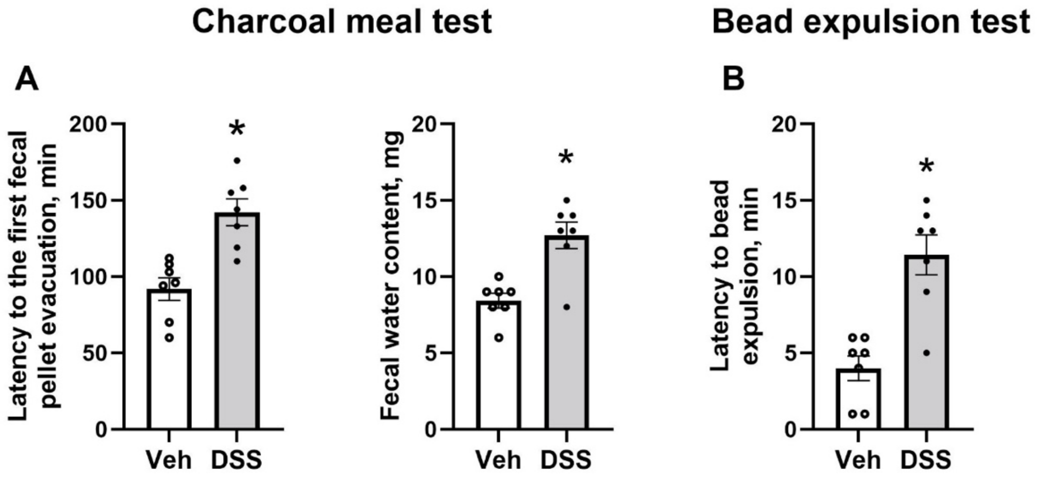

Supplementary Figure S2. Effects of 5% DSS challenge on gastrointestinal function. (A) Whole-gut transit latency and fecal water content in the charcoal meal test. **(B)** Latency in the bead expulsion test. Veh- vehicle-treated group, DSS- 5%DSS-treated group. Data are presented as mean±SEM; individual data points represent individual animals. Two-tailed Student's t-test, *p<0.05. All n=7.

Mice exposed to 5% DDS also demonstrated significantly lower sucrose preference (p=0.0048, t-test). The number of entries and total time spent in open arms of the elevated plus maze did not differ significantly between group (both p>0.05, t-test). In the open field, total time spent in the center was significantly lower in mice exposed to 5% DDS (p<0.0001, t-test), while number of grooming events was significantly elevated in this group (p=0.0002, data not shown).

References

Erben U, Loddenkemper C, Doerfel K, Spieckermann S, Haller D, Heimesaat MM, Zeitz M, Siegmund B, Khl AA. A guide to histomorphological evaluation of intestinal inflammation in mouse models. Int J Clin Exp Pathol. 2014 Jul 15;7(8):4557-76. PMID: 25197329; PMCID: PMC4152019.

Feldman AT, Wolfe D. Tissue processing and hematoxylin and eosin staining. Methods Mol Biol. 2014;1180:31-43. doi: 10.1007/978-1-4939-1050-2_3. PMID: 25015141.
